# High Mdm4 levels suppress p53 activity and enhance its half-life in acute myeloid leukaemia

**DOI:** 10.18632/oncotarget.1559

**Published:** 2013-11-22

**Authors:** Ban Xiong Tan, Kian Hoe Khoo, Tit Meng Lim, David Philip Lane

**Affiliations:** ^1^ p53 Laboratory, A-STAR, Singapore; ^2^ Department of Biological Sciences, National University of Singapore, Singapore

**Keywords:** p53, AML, Mdm4

## Abstract

Although p53 is found mutated in almost 50% of all cancers, p53 mutations in leukaemia are relatively rare. Acute myeloid leukaemia (AML) cells employ other strategies to inactivate their wild type p53 (WTp53), like the overexpression of the p53 negative regulators Mdm2 and Mdm4. As such, AMLs are excellent candidates for therapeutics involving the reactivation of their WTp53 to restrict and destroy cancer cells, and the Mdm2 antagonist nutlin-3 is one such promising agent. Using AML cell lines with WTp53, we identified stable and high levels of p53 in the OCI/AML-2 cell lines. We demonstrate that this nutlin-3 sensitive cell line overexpressed Mdm4 to sequester, stabilise and inhibit p53 in the cytoplasm. We also show that elevated Mdm4 competed with Mdm2-p53 interaction and therefore extended p53 half-life while preventing p53 transcriptional activity. Our results provide biochemical evidence on the dynamics of the p53-Mdm2-Mdm4 interactions in affecting p53 levels and activity, and unlike previously reported findings derived from genetically manipulated systems, AML cells with naturally high levels of Mdm4 remain sensitive to nutlin treatment.

**Key Points:**

Endogenously high levels of Mdm4 inhibit and sequester p53 in AML.

High levels of Mdm4 do not block function of Mdm2 inhibitors in AML.

## INTRODUCTION

The guardian of the genome *TP53* codes for a transcription factor central in responding to a myriad of stress stimuli whose activation results in the induction of several genes to bring about DNA repair, metabolic changes, anti-oxidant responses, cell cycle arrest, apoptosis or senescence [1]. Cancers with aberrations in p53 have either mutated p53, or have dysfunctional p53 regulation. The latter is commonly achieved through Mdm2, which is responsible for nuclear export of p53 [2] and targeting p53 for ubiquitin-mediated proteasomal degradation [3]. Recent evidence implicates Mdm4, an Mdm2 homologue, in the inactivation and degradation of p53 [4]. Despite being highly homologous with Mdm2 and having a C-terminal RING domain, Mdm4 does not have any E3 ligase activity. It is, however, capable in suppressing p53 transcriptional activity by binding the p53 transactivation domain via its N-terminal domain [5, 6]. Also, unlike Mdm2, Mdm4 expression levels are not dependent on p53, though Mdm2 targets Mdm4 and itself for proteasomal degradation [7]. Both Mdm2 and Mdm4 are mutually dependent on each other to bring about effective downregulation of p53 [8]. Mdm4 forms heterodimers with Mdm2 through their RING domains, and this stimulates the Mdm2 E3 ubiquitin ligase activity, heightening polyubiquitination of p53 [9].

Although more than 50% of solid tumours carry *TP53* mutations, *TP53* mutations are rare in leukaemias [10]. Instead, WTp53 in leukaemias is frequently inactivated through abnormalities in Mdm2, and as much as 50% of leukaemias are found to overexpress Mdm2 [11]. This block in p53 signalling contributes greatly to the resistance of leukaemic cells towards apoptosis. An effective therapeutic strategy is the restoration of WTp53 function, through the disruption of its interaction with its negative regulators. The use of nutlin-3, the selective and potent inhibitor of the p53-Mdm2 interaction, in the treatment of WTp53 positive leukaemia is therefore potentially very rewarding [12, 13].

Here, we examined an AML cell line, OCI/AML-2, which harbours high basal levels of WTp53. Using this AML line as a model, we sought to understand the mechanics governing the constant maintenance of a large pool of WTp53 without spontaneously undergoing cell cycle arrest or apoptosis, and demonstrate that the overexpression of Mdm4 is responsible for modulating p53 localisation, half-life and activity. Moreover, unlike previously reported observations, nutlin response is not necessarily limited by the overexpression of Mdm4 in AML cells.

## RESULTS

### AML2 cells are sensitive to nutlin-3, despite high basal levels of WTp53

**Figure 1:**
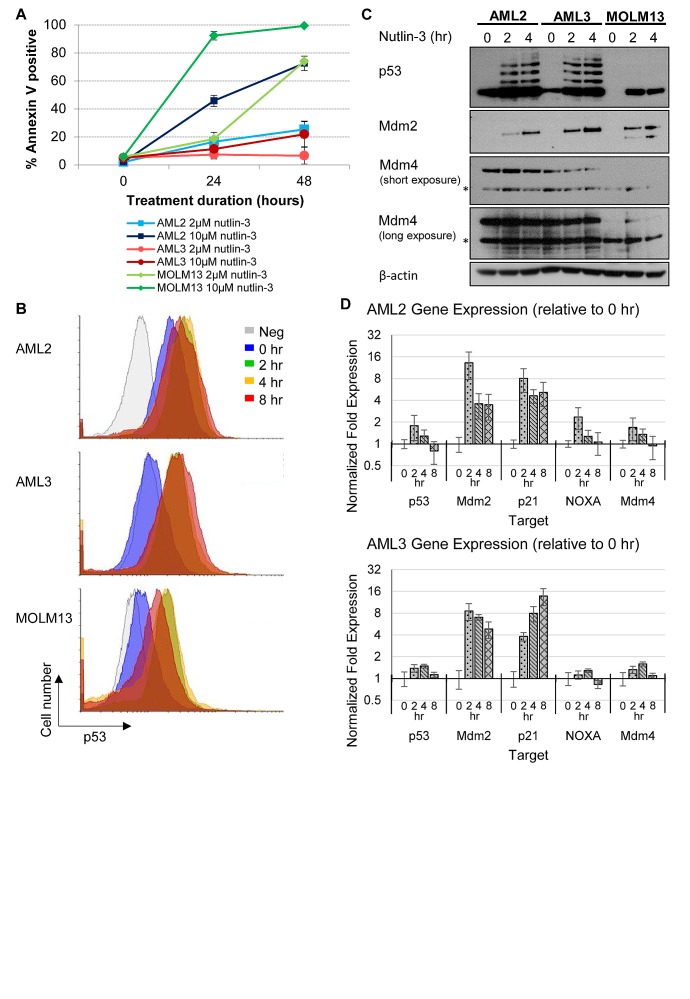
Differential p53 and apoptotic response of AML cell lines towards nutlin-3, with AML2 cells having high basal p53 levels (A) AML2, AML3 and MOLM13 cells were treated with 2μM or 10μM nutlin-3 for 24 or 48 hours. The cells were stained with Annexin V-Cy5 and analysed by flow cytometry. (B) AML2, AML3 and MOLM13 cells treated with 10μM nutlin-3 for 0, 2, 4, and 8 hours were stained with mouse anti-p53 (DO-1) primary antibodies (or mouse normal IgG as a negative control) and goat anti-mouse AlexaFluor488 secondary antibodies. The p53 levels in the cells were then analysed by flow cytometry. (C) AML2, AML3 and MOLM13 cells were treated with 10μM nutlin-3 for the indicated durations and were lysed and immunoblotted with the respective antibodies. * indicates non-specific bands. (D) RNA was harvested from AML2 and AML3 cells treated with 10μM nutlin-3 for 0, 2, 4, or 8 hours and reversed transcribed for quantitative PCR for the indicated genes

We looked into the effects of nutlin-3 on AML cells by testing the sensitivities of three AML cell lines OCI/AML-2 (AML2), OCI/AML-3 (AML3) and MOLM13. All three cell lines were established from the peripheral blood of AML patients and are wild type for p53. A notable difference is that AML3 cells harbour the cytoplasmic mutant NPM, while AML2 and MOLM13 cells are wild type for NPM [15, 16]. AML2 and AML3 cells also carry *DNMT3A* mutations [17]. Apoptosis was assayed by staining nutlin-3 treated cells with Annexin V and analysing them using flow cytometry (Figure 1A). MOLM13 cells were extremely sensitive to nutlin-3, with almost all cells (92.3% and 99.2% after 24 and 48 hours) undergoing apoptosis with treatment of 10μM nutlin-3. AML2 cells were less sensitive compared to MOLM13 cells, exhibiting significant cell death after treatment with 10μM nutlin-3 (45.8% and 72.6% after 24 and 48 hours). However, AML3 cells showed resistance towards nutlin-3, exhibiting only a relatively small percentage of apoptotic cells at 10μM nutlin-3 (11.3% and 21.9% after 24 and 48 hours), a rate lower than that achieved in AML2 cells treated with only 2μM nutlin-3 (16.5% and 25.3% after 24 and 48 hours). Thus despite having WTp53, the three cell lines responded differently to nutlin-3 treatment.

In order to understand this discrepancy, we examined the effect of nutlin-3 on p53 and p53-regulated expression. Flow cytometry of p53 staining in the three AML cell lines showed a higher level of p53 in untreated AML2 cells, with p53 levels increasing even higher after nutlin-3 exposure (Figure 1B, 1C). In contrast, AML3 and MOLM-13 displayed low basal levels of p53, only increasing upon nutlin-3 induction. It is noted that in AML2 and AML3 induction by nutlin-3 results in ubiquitinated species of p53, concordant with previously reported findings [18]. Interestingly, while high levels of WTp53 are normally associated with cell cycle arrest and apoptosis, the high basal levels of p53 observed in untreated AML2 cells did not result in cell cycle arrest or apoptosis.

To ensure that the p53 in these AML cell lines was wild type and functional, the three cell lines were treated with nutlin-3 and Mdm2 protein upregulation, an indication of p53 activity, was observed (Figure 1C). In addition, p53-regulated genes like Mdm2, p21 and NOXA were upregulated at the RNA level in response to nutlin-3 induction in AML2 and AML3 cells (Figure 1D). The abnormal level and activity of p53 in AML2 cells could be a result of aberrations in p53 negative regulators, chief among them are Mdm2 and Mdm4. Sequencing of cDNA from AML2 cells revealed that there were no mutations in either Mdm2 or Mdm4 (data not shown). To look for other means of Mdm2 deregulation, we examined the effect of ATM kinase on Mdm2, where ATM kinase-mediated phosphorylation of S395 inhibits the Mdm2-mediated degradation of p53 [19]. We used the ATM inhibitor (ATMi) KU-55933 (Calbiochem, USA) to see if the high level of p53 is due to ATM kinase activity. Although treatment with the ATMi downregulated p53 levels in AML3 cells, the p53 levels in AML2 were largely unchanged (Figure S1).

Similarly, we examined another critical regulator of Mdm2, the tumour suppressor ARF. ARF binds to the acidic region of Mdm2 and inhibits p53 ubiquitination and degradation [20]. However, there are no significant differences in ARF expression between AML2 and AML3, indicating that ARF is probably not responsible for the accumulation of p53 (Figure S1). In addition, we looked into a few of the ubiquitin specific proteases (USPs) that are known to affect p53. USP5 deubiquitinates p53 [21], while USP2a acts on Mdm2 [22], and differences in the levels of the two proteases can affect p53 levels. There were no significant differences in the levels of these two USPs between AML2 and AML3 cells, indicating that they are not involved in mediating p53 accumulation (Figure S1).

Mdm2 levels and response in AML2 cells were similar to that seen in AML3 cells, albeit at a lower level (Figure 1C). However, unlike Mdm2, the Mdm4 levels in AML2 cells were much greater than in AML3 cells, and remained high upon nutlin treatment (Figure 1C). Comparatively, the Mdm4 levels in AML3 were lower and dropped rapidly upon drug treatment. (Figure 1C). Given that Mdm4 is known to bind and sequester p53 [23], and that the overexpression of Mdm4 is exploited by several cancers to deregulate p53 function [24-26], the high level of Mdm4 is a likely candidate responsible for the high basal p53 in AML2.

### WTp53 in AML2 is stable but inactive at physiological conditions

**Figure 2:**
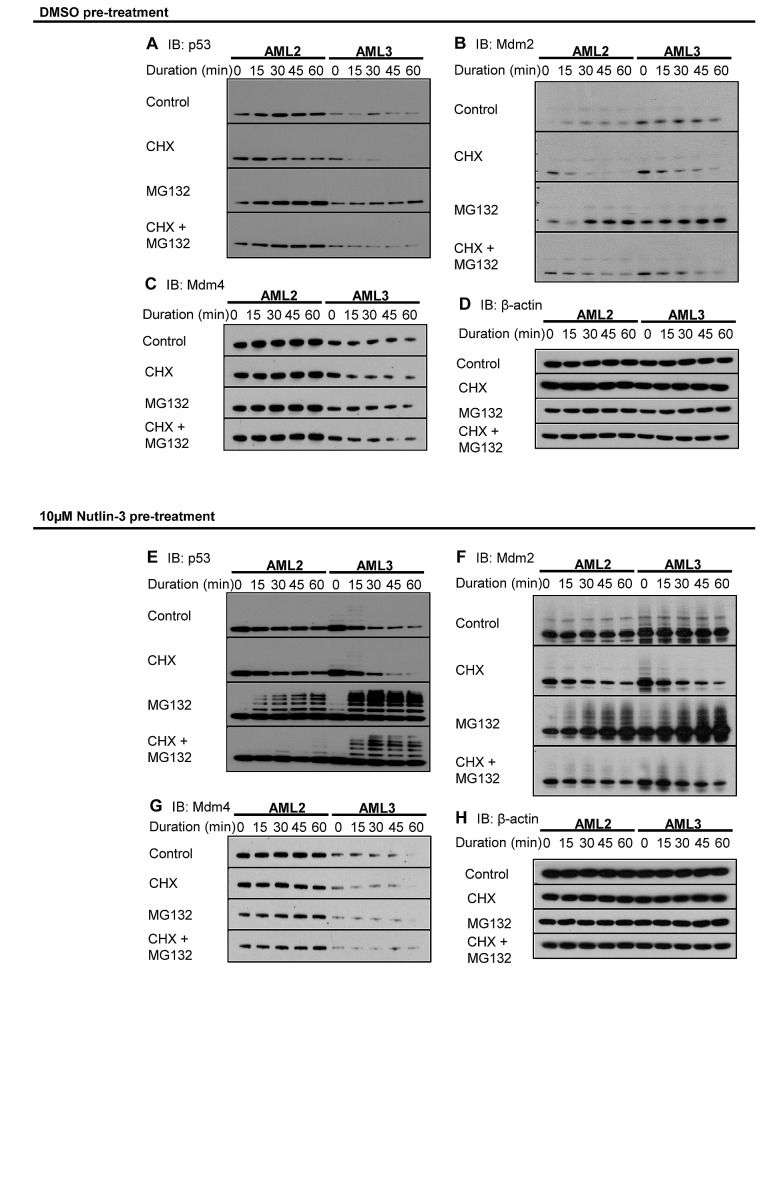
Excessive Mdm4 correlates to increased p53 stability in AML2. AML2 or AML3 cells were treated with 10μM nutlin-3 or DMSO for 4 hours before replacing with fresh media supplemented with DMSO, 10μg/ml cycloheximide (CHX), 10μM MG132, or both CHX and MG132. Cells were harvested every 15 minutes and immediately lysed in 95°C SDS lysis buffer. The lysates were sonicated, separated in SDS-PAGE, and immunoblotted for (A, E) p53, (B, F) Mdm2, (C, G) Mdm4 and (D, H) β-actin.

To understand the mechanism by which WTp53 in AML2 cells are elevated at a basal level, we tracked the amounts of p53, Mdm2 and Mdm4 present in both AML2 and AML3 cells at different intervals. The inhibitor of translation, cycloheximide (CHX) and the proteasome inhibitor MG132 were used to offer insights on the half-life and ubiquitination of the proteins respectively. Compared to AML3, the p53 in AML2 was not only more abundant, but also exhibited a longer half-life, evident after CHX treatment (Figure 2A). Further, there was a greater amount of accumulation after proteasomal inhibition, indicating that there was a higher basal level of expression compared to AML3, where p53 hardly accumulated. The combination of CHX and MG132 accurately showed the steady state level of p53 in the two cell lines, potentially ruling out the involvement of other non-proteasomal degradation pathways.

There were little differences between the two cell lines in terms of Mdm2 levels and stability (Figure 2B). At basal level, both AML2 and AML3 expressed low levels of Mdm2, given the Mdm2-p53 negative feedback loop. However, there were marked differences when comparing Mdm4 in the two cell lines (Figure 2C). Although both cell types expressed stable levels of Mdm4, the levels in AML2 were significantly higher than that in AML3. This reinforced the observation of elevated Mdm4 in AML2 cells. β-actin was used as a loading control (Figure 2D).

A measure of p53 stability and Mdm2/Mdm4 activity can be observed by pre-treatment and withdrawal of nutlin-3 [18]. CHX and MG132 were added to the cells after nutlin-3 withdrawal to chart degradation and accumulation. Cells treated with the Mdm2 antagonist accumulate p53 and p53-responsive gene products rapidly, and the withdrawal of nutlin-3 reverses the upregulation, resulting in rapid degradation. Treatment with nutlin-3 resulted in massive upregulation of p53, but, unlike in AML3, the p53 in AML2 was sustained at a high level (Figure 2E). This observation was emphasised with the use of CHX to inhibit protein synthesis, with p53 in AML3 diminishing rapidly, while the p53 in AML2 remained high. The use of MG132 also showed that p53 in AML3 cells was highly ubiquitinated, exhibiting greater levels of polyubiquitinated species of p53 compared to the p53 in AML2. This would explain the rapid loss of p53 in AML3 cells, as polyubiquitinated p53 would be rapidly degraded in proteasomes. Interestingly, the combination of CHX and MG132 suggested that only newly synthesised p53 underwent ubiquitination. This implies that there was a static pool of p53 in AML2 that was resistant to degradation.

Mdm2 in both cell lines behaved as expected after nutlin-3 withdrawal (Figure 2F). The half-lives of Mdm2 in both AML2 and AML3 were similar, and the level of ubiquitination were comparable. However, the levels of Mdm4 between the two cell lines were different, with Mdm4 in AML3 being rapidly degraded (Figure 2G). On the other hand, Mdm4 levels in AML2 remained steady throughout the entire interval, even in the presence of CHX and/or MG132. Figure 2H shows the β-actin loading control.

### Stability of p53 is determined by ratio of p53-Mdm2 to p53-Mdm4 interactions

**Figure 3:**
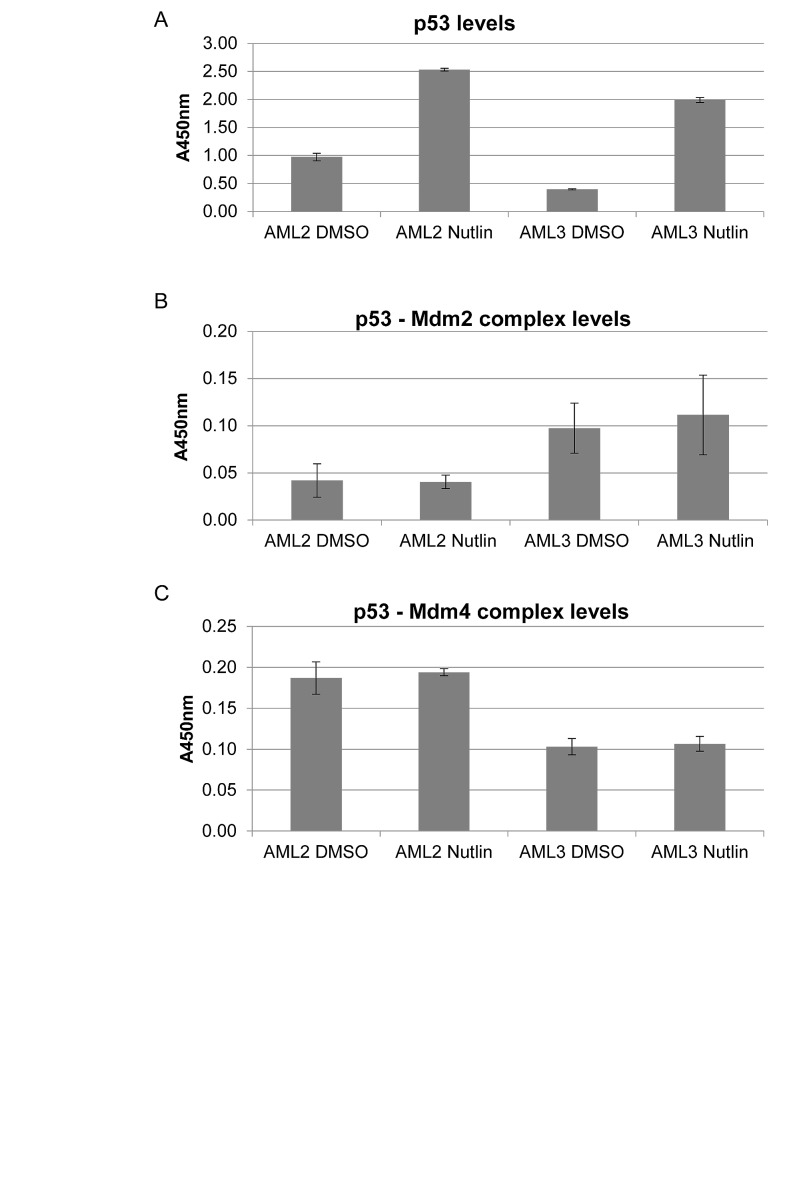
p53 in AML2 binds preferentially to Mdm4 rather than Mdm2, with or without nutlin-3 treatment AML2 or AML3 cells were treated with 10μM nutlin-3 or DMSO for 4 hours being harvested for cell lysis. 6mg/ml cell lysates were added to ELISA plates coated with anti-p53 (Bp53 10.1) antibodies. Detection of the indicated proteins complexed with captured p53 was achieved using antibodies (HRP conjugated or with HRP conjugated secondary antibodies) specific to p53 (A), Mdm2 (B) and Mdm4 (C). The graphs depict the mean absorbance of three independent experiments with 95% confidence interval

In order to determine whether the stability of p53 in AML2 was conferred by sequestration by high levels of Mdm4, we sought to quantify the interactions between p53 and Mdm2, and p53 and Mdm4. An ELISA using immobilised anti-p53 (Bp53 10.1) antibodies was used, recognising and binding p53 at the C-terminus, away from the Mdm2/Mdm4 binding site. Captured complexes in cell lysates from AML2 or AML3 cells treated or untreated with nutlin-3 were detected using a sandwich assay. As expected, the amount of p53 captured in the assay corresponded to the amount of p53 in each cell line, with high basal levels of p53 in AML2 and low levels in AML3, and both increased in response to nutlin-3 treatment (Figure 3A). The ELISA also showed that the p53 in AML3 cells was mostly complexed to Mdm2 (Figure 3B), while the p53 in AML2 cells was mostly bound to Mdm4 (Figure 3C). This suggested that while p53 in AML3 had the propensity to be bound to Mdm2 and therefore subsequently degraded, p53 in AML2 was bound to Mdm4 instead, and this interaction prevents p53 degradation as well as p53 activity.

**Figure 4:**
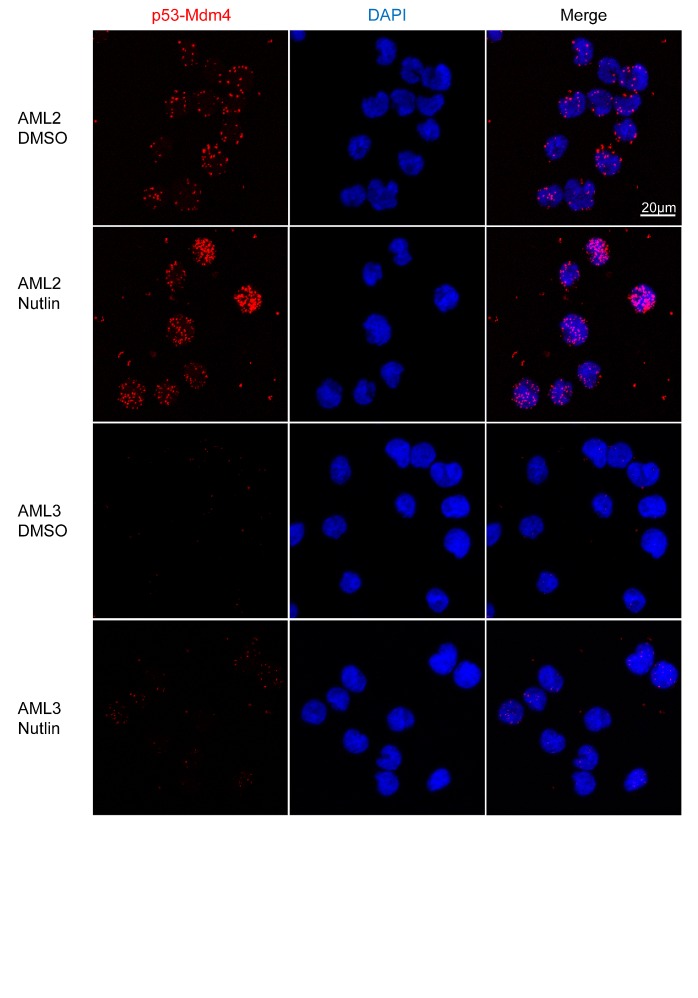
p53 in AML2 binds to Mdm4, with or without nutlin-3 treatment. AML2, AML3 or MOLM13 cells were treated with 10μM nutlin-3 or DMSO for 4 hours fixation and permeabilisation. PLISA was performed using the Duolink kit according to manufacturer's recommendations and visualised with confocal microscopy. Micrographs show the maximum intensity projection of 13 z-stacks, each 0.49μm thick. Each red spot represents an interaction between p53 and Mdm4

To reaffirm the findings of a higher level of p53-Mdm4 interaction in AML2, a proximity ligation *in situ* assay (PLISA) for the interaction was performed (Figure 4). As each PLISA spot corresponds to an interaction event, there was a greater amount of p53-Mdm4 interaction in AML2, with or without nutlin-3 treatment. In contrast, the p53-Mdm4 interaction in AML3 was exceedingly low, and only slightly increased in response to nutlin-3 treatment. It is interesting to note that in AML2, most of the interactions occur outside the nucleus in the cytoplasm. This observation reinforces the hypothesis that Mdm4 sequesters and inactivates p53 by localising it in the cytoplasm, as observed by the overexpression of nuclear localisation mutants of Mdm4 [23].

### Reduction of Mdm4 caused p53 reactivation in AML2

**Figure 5:**
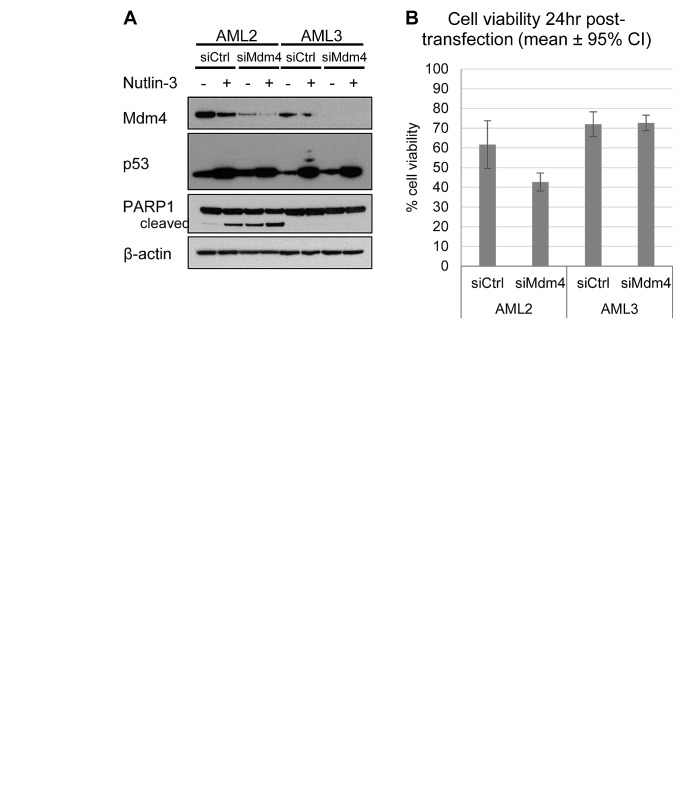
Knockdown of Mdm4 induced cell death in AML2, but not in AML3 (A) AML2 or AML3 cells transfected with non-targeting siRNA (siCtrl) or siRNA against Mdm4 (siMdm4) for 24hr before treatment with 10μM nutlin-3 (or DMSO) for 4hr, and blotted for the indicated proteins. (B) Cell viability of AML2 and AML3 cells 24hr post-transfection with siCtrl or siMdm4, as assayed by PI exclusion.

To test the hypothesis that Mdm4 is responsible for the accumulation and inactivation of p53 in AML2 cells, siRNA against Mdm4 (siMdm4) was employed. Figure 5A demonstrates the successful knockdown of Mdm4 in AML2 and AML3 cells transfected with siMdm4, compared to the control using a non-targeting siRNA (siCtrl). Compared to AML3 cells, siMdm4-transfected AML2 cells displayed a decrease in cell viability 24 hours post-transfection, indicating that Mdm4 is required to prevent cell death in AML2 (Figure 5B). This drop in cell viability corresponded to an increase in PARP cleavage (Figure 5A), implying caspase-mediated apoptosis. Put together, the results suggest a reduction of the high levels of Mdm4 brought about the prevention of p53 suppression in AML2, leading to apoptotic signalling and cell death.

**Figure 6:**
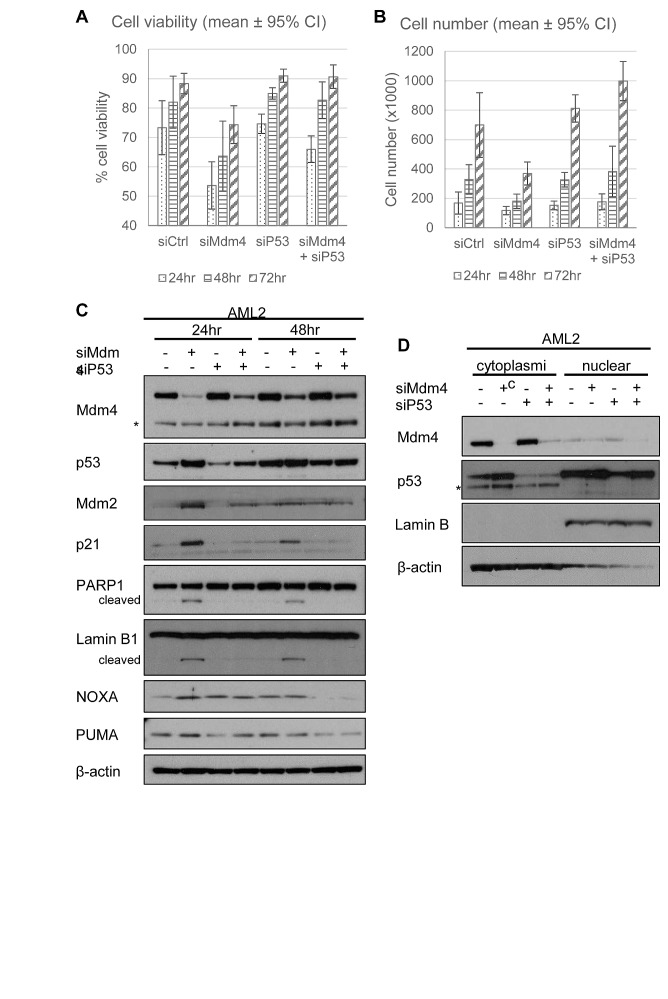
Knockdown of p53 rescues Mdm4 depletion-induced cell death in AML2. AML2 cells were transfected with siCtrl or siMdm4 and/or siP53 and (A) cell viability and (B) cell numbers were analysed 24, 48 and 72 hours post-transfection. Similarly treated AML2 cells were lysed (C) or separated into cytoplasmic or nuclear fractions (D) and analysed for the indicated proteins by Western blot. * indicates non-specific bands.

In order to ascertain that p53 was involved in the loss in cell viability following Mdm4 knockdown, siRNA against p53 (siP53) was co-transfected into AML2 cells along with siMdm4. While siMdm4 resulted in the loss of cell viability and decreased proliferation, the concurrent knockdown of p53 abolished the effects mediated by knocking down Mdm4 (Figure 6A and 6B). Closer examination of the molecular changes following siMdm4 revealed that the reduction of Mdm4 resulted in an increase in p53 levels as well as p53 transcriptional activity, demonstrated by an increase in the protein levels of the p53-transcribed genes *mdm2*, *p21*, *NOXA* and *PUMA* (Figure 6C). This increase in p53 activity was accompanied by the presence of PARP and Lamin B cleavage, both of which indicate apoptosis. These observations were abrogated with the concurrent knockdown of p53, suggesting that p53 was involved in mediating cell death induced by Mdm4 reduction. Subcellular fractionation of AML2 cells indicated that depletion of Mdm4 by siRNA resulted in the increase in p53 in both the cytoplasm and nucleus (Figure 6D), suggesting that p53 could mediate apoptosis in both a transcription dependent or independent manner. These findings strongly suggest that Mdm4 is involved in preventing p53 activation and that upon the removal of Mdm4, p53 becomes active in causing cell death.

## DISCUSSION

Cancers that retain WTp53 often carry mutations in regulatory genes that dampen p53 activity, effectively creating phenotypes similar to p53 mutations. Many of these mutations affect the stability and degradation of p53, governing the amount and/or localisation of p53 in the cancer cell. These mechanisms include Mdm2 overexpression [27], ARF ablation [28], loss of PTEN expression [29], expression of HPV E6 protein in cervical cancers [30], or p53 cytoplasmic sequestration [31]. Here, we show that a leukaemia cell line, AML2, carries high basal levels of WTp53, and an amplified level of Mdm4 effectively binds to and inhibits p53 activity.

High p53 levels in cells are frequently associated with cell cycle inhibition and apoptosis, and it would be seemingly paradoxical to observe high WTp53 levels in actively dividing cancer cells. However, high WTp53 expression levels have been observed in melanomas [32] and testicular cancers [33], and here, we report that a high level of WTp53 is also detected in AML, as demonstrated in the AML2 cell line.

Further investigation of the p53 in AML2 revealed that it is mostly cytoplasmic and bound to Mdm4. While Mdm4, together with Mdm2, is responsible for the ubiquitination and subsequent degradation of p53 [34], Mdm4 overexpression was previously reported to inhibit p53 activity by direct binding, sequestering the tumour suppressor in the cytoplasm, outcompeting Mdm2-p53 interaction and protecting p53 against proteasomal degradation [5, 23, 35, 36]. This coincides with the observations reported here that p53 was kept inactive in the cytoplasm at high levels by interactions with Mdm4. Although high Mdm4 levels have been reported in melanomas[26], retinoblastoma [25], head and neck squamous cancers [37], the high levels of both p53 and Mdm4 have not been described until now. In fact, Mdm4 has been reported to be amplified in a panel of leukemia cell lines (Figure S2) [38]. Also, unlike in systems where Mdm4 had been exogenously introduced [39-41], here we demonstrate that endogenously high levels of Mdm4 can effectively inhibit p53 activity and sequester it in the cytoplasm.

In the pulse chase experiments, it was observed that only nascent p53 was ubiquitinated in AML2 cells (Figure 5.6E). As p53 in AML2 was mostly bound to Mdm4 but not Mdm2, the lack of p53 ubiquitination could be due to Mdm4 out-competing Mdm2 for p53 binding, preventing Mdm2 from ubiquitinating p53. This would also explain the increased stability and longer half-life of p53 observed in AML2 cells. Similarly, the high levels of Mdm4 would also prevent Mdm2-mediated ubiquitination and subsequent proteasomal degradation of Mdm4, allowing the accumulation of Mdm4 in AML2 cells. These observations highlight the importance of the stoichiometry of p53, Mdm2 and Mdm4 in maintaining their stability and degradation.

Unlike in model studies where Mdm4 overexpression blocked nutlin-induced apoptosis [39, 40], the high levels of Mdm4 did not confer much resistance in AML2 cells. AML2 cells, while being more sensitive than AML3 cells, did not exhibit rapid cell death compared to MOLM13 cells. It is speculated that nutlin-3 prevents the degradation of newly-synthesised p53, thereby allowing the accumulation of p53 beyond what can be sequestered by Mdm4. Nonetheless, the loss of cell viability in response to the knockdown of Mdm4 indicated that Mdm4 did indeed prevent cell death in AML2 cells. Since the precarious balance of Mdm2 and Mdm4 determines p53 stability and activity, it is feasible that disruption of Mdm2-p53 interaction by nutlin-3, in a cell ‘addicted’ to Mdm4 as an oncogene, was sufficient to increase p53 levels beyond what can be sequestered by overexpressed Mdm4, leading to apoptosis.

The knockdown of Mdm4 by siRNA in AML2 cells resulted in cell death characterised by PARP cleavage. p53 nuclear accumulation was observed and p53-dependent genes were upregulated, suggesting that the release of p53 from its inhibition by Mdm4 led to cell death in a p53 dependent manner. Canonically, p53-mediated apoptosis requires the transcriptional activation of pro-apoptotic genes like PUMA, NOXA and Bax. However, recent evidence implicate that the stress-induced cytoplasmic and mitochondrial accumulation of p53 can lead to the direct activation of Bax and/or Bak [42, 43]. These pro-apoptotic members of the Bcl-2 family oligomerise and form pores in the mitochondria outer membrane, triggering the intrinsic apoptotic caspase cascade. This p53-dependent role in apoptosis was found to be present when p53 nuclear translocation was prevented [42] and when transcription was inhibited [44], and the activation of pro-apoptotic proteins by p53 was found to precede p53-activated transcription [45]. Further investigation led to the discovery that p53 can bind Bcl-2 [46] and Bcl-xL [47], preventing their anti-apoptotic activities. The findings of increased cytoplasmic p53 levels upon siMdm4 suggest the possibility of a transcription independent role of p53 in mediating apoptosis in AML2 cells.

Anti-leukaemia chemotherapeutic drugs like AraC, etoposide, daunorubicin and doxorubicin traditionally target DNA replication because of the rapid proliferation of leukaemia cells. However, these drugs also adversely affect numerous other rapidly dividing cell types, forming the basis of detrimental side effects. Treatment of leukaemia therefore is a balance of destroying the majority of leukaemic cells while killing as few normal cells as possible. These drugs are also genotoxic in nature, introducing the possibility of inducing further mutations that potentially generate other cancers, or even result in the induction of chemoresistance in the existing one. In fact, a major cause of AML is the use of cytotoxic drugs previously used to treat other forms of cancer. As a specific inhibitor of Mdm2-p53 interaction, nutlin-3 offers advantages over traditional chemotherapeutic drugs in that, at optimal doses, its apoptotic effect is selective for tumour cells while normal cells undergo reversible cell cycle arrest [48]. Moreover, since the mechanism of action does not involve DNA intercalation or damage, nutlin-3 is non-genotoxic [49]. Hence, the use of nutlin-3 in a WTp53 cancer like AML would be extremely suitable.

The differential response of AML cells to nutlin-3 demonstrates the need to understand the underlying causes of resistance. Optimised nutlin analogues are currently undergoing clinical trials, and in light of the data presented here, cancers refractory to nutlin-treatment should not be dismissed entirely without further investigation. Given that there is evidence of Mdm4 overexpression in AML, combinatorial treatments with Mdm2 and Mdm4 inhibitors would allow lowered dosages and even fewer adverse effects.

## MATERIALS AND METHODS

### Cell culture and siRNA transection

AML cell lines OCI/AML-2, OCI/AML-3 and MOLM13 were cultured in Alpha Minimum Essential Media supplemented with 20% foetal calf serum and 100U/ml penicillin and 100μg/ml streptomycin, as previously described [14]. The Nucleofector system (Amaxa; programme X-001) was used to transfect 2.5 x 10^6^ cells with 1μM pooled non-targeting siRNA (siCtrl), siRNA against Mdm4 (siMdm4) and/or siRNA against p53 (siP53) (Thermo Scientific). Cell viability and cell number were assessed using the Adam automated cell counter (Digital Bio).

### Flow cytometry

For apoptosis assays, 2.5 x 10^5^ cells were resuspended in binding buffer (10mM HEPES pH 7.4, 140mM NaCl, 2.5mM CaCl_2_) with 1μl Annexin V-Cy5 (BioVision). After 15 minutes incubation at room temperature, the cells were pelleted and resuspended again in binding buffer for analysis. For immunostaining assays, 2.5 x 10^5^ cells were fixed and permeabilised in ice-cold 70% ethanol for 30 minutes at −20°C. The cells were washed with PBS and stained with 1μg mouse anti-p53 (DO-1) or mouse isotype control antibody, washed thrice with PBS and stained with 1μg goat anti-mouse Alexa488 (Invitrogen), and washed thrice again. Flow cytometry analysis on single cells was performed with BD LSR II (BD Biosciences). Data analysis was performed using Flowing Software 2 (Turku Centre for Biotechnology).

### Western blotting

Lysates were prepared by lysing cells in 95°C SDS lysis buffer (20mM Tris Cl pH 8.0, 2% SDS, 10% glycerol), and briefly sonicated to shear DNA. Equal amounts of protein measured by BCA quantification (Pierce), were loaded in SDS-PAGE gels and transferred onto nitrocellulose with the iBlot system (Invitrogen). The primary antibodies used were: mouse anti-p53 (DO-1; 1:5000), mouse anti-Mdm2 (2A9; 1:5000), rabbit anti-Mdm4 (Bethyl; 1:5000), mouse anti-p21 (118; 1:2000), mouse anti-NOXA (Calbiochem), rabbit anti-PUMA (Calbiochem), rabbit anti-PARP1 (Cell Signaling Technologies; 1:2000), rabbit anti Lamin B1 (Cell Signaling Technologies; 1:2000), mouse anti-β-actin (AC15; Sigma; 1:10 000), and mouse anti-GAPDH (Ambion; 1:10 000). Secondary anti-mouse or -rabbit antibodies conjugated to HRP (Dako; 1:10 000) were detected using SuperSignal West Dura chemiluminescent kit (Pierce) exposed on X-ray film (Fuji).

### Quantitative real time PCR

Total RNA was extracted from AML2 and AML3 cells with a Qiagen RNAeasy Kit. cDNA was obtained by reverse transcription using random hexamers and 1μl (5% of the reaction) was used as the template for subsequent real time PCR with 2X iQ Supermix (Biorad) and 1μM forward and reverse primers (Table 1). The reactions were performed in triplicate in a 384-well format with 10μl reaction volume. The melt curve for each primer set was verified to have a single distinct peak and gel electrophoresis of the PCR products only produced one clear band.

**Table 1:** Primers used for quantitative real time P

**p53**	Forward Reverse	5' – TAAAAGATGTTTTGAATG – 3' 5' – ATGTGTGTGATGTTGTAGATG – 3'
**Mdm2**	Forward Reverse	5' – GAGATGGTTAGAAAAG – 3' 5' – GATGGATTGATGGGT – 3'
**p21**	Forward Reverse	5' – ATAGAGGAGGGATGT – 3' 5' – AGGGAAGTATA – 3'
**Noxa**	Forward Reverse	5' – GTGTTGGAAAGGAAGA – 3' 5' – AGGAGTTAATA – 3'
**Mdm4**	Forward Reverse	5' – GAAGAAATTTAATTAAGAA – 3' 5' – TTTGAAAATTGAATAAATTT – 3'
**GAPDH**	Forward Reverse	5' – GATTTTTTTGGTG – 3' 5' – TGAAATGAGAGTT – 3'

### Enzyme-linked immunosorbent assay (ELISA)

20μg/ml mouse anti-p53 (Bp53 10.1) was immobilised onto high binding capacity polystyrene 96-well plates (Corning) and incubated with lysates from 2 x 10^7^ cells (lysis buffer: 50mM Tris Cl pH 8.0, 150mM NaCl, 10mM EDTA, 0.1% NP-40, 5mM NaF, 200μM Na_3_VO_4_, 1mM dithiothreitol, 1x protease inhibitor cocktail (Roche)). After washing with PBS, protein complexes were detected using HRP-conjugated mouse anti-Mdm2 (2A9), HRP-conjugated mouse anti-p53 (DO-1), or rabbit anti-Mdm4 (Bethyl) with secondary swine anti-rabbit HRP antibodies and TMB substrate (Bio-Rad). The reaction was stopped with 1.0M H_2_SO_4_ and the absorbance at 450nm was measured using an EnVision Plate Reader (Perkin Elmer).

### Proximity Ligation *In Situ* Assay (PLISA)

PLISA was performed using the Duolink *In Situ* Kit (OLink Biosciences, Sweden) according to manufacturer's protocols. Cells smeared onto Polysine (Thermo Scientific) glass slides were fixed with 3.7% paraformaldehyde in PBS and permeabilised with 0.2% Triton X-100 in PBS, blocked and incubated with 5 μg/ml of the following antibodies: mouse anti-p53 (Bp53 10.1) and rabbit anti-Mdm4 (Bethyl), or mouse anti-Mdm2 (2A9) and rabbit anti-p53 (CM-1). Images comprising 30 0.49μm-thick z-stacks were obtained with a Zeiss LSM510 confocal microscope with a 40X 1.3 NA objective.

## SUPPLEMENTARY FIGURES


