# Supplementary material for: High Mdm4 levels suppress p53 activity and enhance its half-life in acute myeloid leukaemia
Source: Oncotarget. 2013 Nov 22;5(4):933–43. doi: 10.18632/oncotarget.1559 (PMC4011595; doi:10.18632/oncotarget.1559)
Supplement: Supplementary file 1 [file oncotarget-05-933-s001.pdf]

High Mdm4 levels suppress p53 activity and enhance its half-life in acute myeloid leukaemia - Tan et al

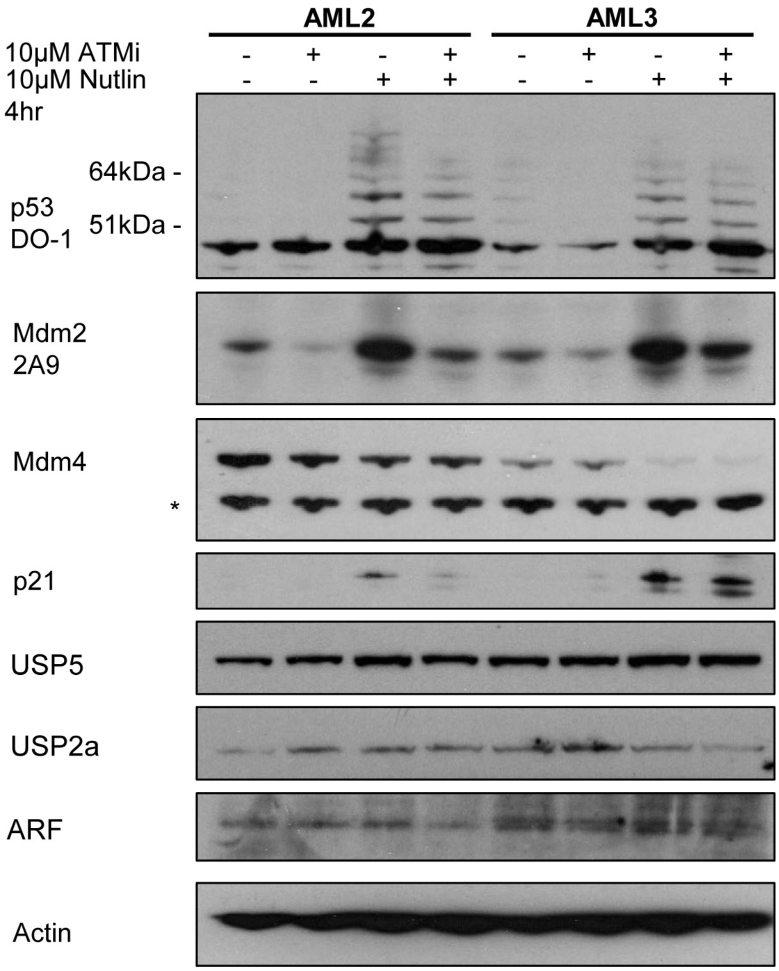

Figure S1: High levels of p53 in AML2 were not due to ATM kinase activity, overexpression of USPs, or ARF. AML2 and AML3 cells were treated with 10µM nutlin-3 for the indicated durations in the presence or absence of ATM kinase inhibitor (ATMi) KU55933 for 4 hours. The cells were lysed and immunoblotted with the respective antibodies. * indicates non-specific bands

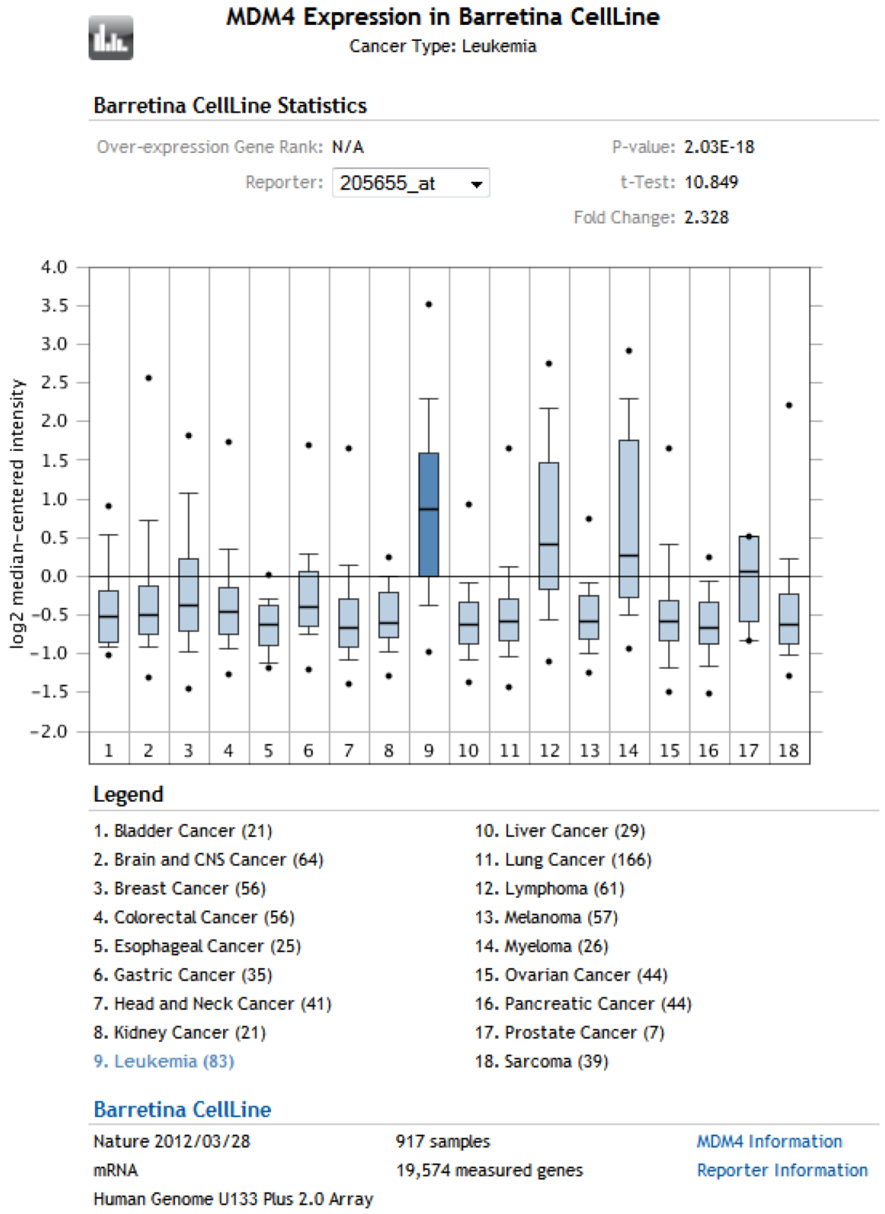

Figure S2: Leukaemia cells overexpress Mdm4. Mdm4 mRNA expression levels were obtained from a study by Barretina et al. [38] involving a panel of 947 human cancer cell lines using Oncomine (www.oncomine.org).
